# Differential circulating concentrations of adipokines, glucagon and adropin in a clinical population of lean, overweight and diabetic cats

**DOI:** 10.1186/s12917-017-1011-x

**Published:** 2017-04-04

**Authors:** Rizaldy C. Zapata, Melissa D. Meachem, Natalia Cavalca Cardoso, Susan O. Mehain, Chantal J. McMillan, Elisabeth R. Snead, Prasanth K. Chelikani

**Affiliations:** 1 Department of Production Animal Health, University of Calgary, 3280 Hospital Drive NW, Calgary, AB T2N4N1 Canada; 2 Department of Veterinary Pathology, University of Saskatchewan, 52 Campus Drive, Saskatoon, SK Canada; 3 Department of Small Animal Clinical Sciences, Western College of Veterinary Medicine, University of Saskatchewan, 52 Campus Drive, Saskatoon, SK Canada; 4 Veterinary Teaching Hospital, College of Veterinary Medicine, Washington State University, 205 Ott Rd, Pullman, WA 99164-7060 USA; 5 Department of Veterinary Clinical and Diagnostic Sciences, Faculty of Veterinary Medicine, University of Calgary, 3280 Hospital Dr. NW, Calgary, AB Canada

**Keywords:** Feline diabetes, Obesity, Adipokines, Glucagon, Adropin

## Abstract

**Background:**

Dyslipidemia, dysregulated adipokine secretion and alteration in glucagon and adropin concentrations are important obesity-related factors in the pathophysiology of human Type 2 diabetes; however, their roles in the pathophysiology of feline diabetes mellitus are relatively unknown. Here, we determined the concentrations of circulating leptin, adiponectin, pro-inflammatory cytokines, glucagon, adropin, triglycerides, and cholesterol, in non-diabetic lean and overweight cats and newly diagnosed diabetic cats. Client-owned cats were recruited and assigned into 3 study groups: lean, overweight and diabetic. Fasting blood samples were analyzed in lean, overweight and diabetic cats at baseline and 4 weeks after consumption of high protein/low carbohydrate standardized diet.

**Results:**

Serum concentrations of triglycerides were greater in diabetics at baseline and were increased in both diabetic and overweight cats at 4 weeks. Plasma leptin concentrations were greater in diabetic and overweight at baseline and 4 weeks, whereas adiponectin was lower in diabetics compared to lean and overweight cats at baseline and 4 weeks. Diabetics had greater baseline plasma glucagon concentrations compared to lean, lower adropin than overweight at 4 weeks, and lower IL-12 concentrations at 4 weeks than baseline.

**Conclusions:**

Our results suggest that feline obesity and diabetes mellitus are characterized by hypertriglyceridemia and hyperleptinemia; however, diabetic cats have significantly lower adiponectin and adropin compared to overweight cats. Thus, despite having similar body condition, overweight and diabetic cats have differential circulating concentrations of adiponectin and adropin.

## Background

Diabetes mellitus is a common endocrinopathy in cats [1–3]; however, its pathophysiology is not completely understood. Similar to humans with Type 2 diabetes mellitus (T2DM), obesity is a common clinical feature of feline diabetes mellitus (FDM) [4–6]. Increasing body weight has clearly been shown to decrease insulin sensitivity in cats supporting the important link between obesity and glucose homeostasis [7, 8]. Dyslipidemia, dysregulated adipokine secretions and alteration in glucagon and adropin concentrations are important obesity-related factors in the pathophysiology of human T2DM [9, 10]; however, their roles in the pathophysiology of FDM are relatively unknown [11].

Adipose tissue is a source of hormones and cytokines that modulate energy balance and glucose metabolism. There is substantial evidence that obese and insulin-resistant cats have increased circulating concentrations of leptin when compared to healthy lean cats [12, 13]. However, the association of adiponectin with obesity is inconsistent in felines with some studies indicating a negative correlation [14–18] and others failing to detect a relationship between total circulating adiponectin and obesity [19, 20]. In addition, the adipose transcript abundance of pro-inflammatory cytokines (eg., tumor necrosis factor-α, interferon-ϒ, monocyte chemoattractant protein-1) has been reported to be greater in obese compared to lean cats [21–23]. However, less is known of whether circulating concentrations of these inflammatory mediators are altered with adiposity and diabetes in cats. Moreover, obese cats have increased plasma triglyceride concentrations [24] and higher cholesterol concentrations have been associated with a decreased likelihood of diabetic remission in FDM [25].

The role of gut hormones in the pathophysiology of feline obesity and diabetes is poorly understood. We recently reported that plasma concentrations of the gut hormone glucagon-like peptide-1 (GLP-1) are increased after a meal in cats, with diabetic cats having greater circulating concentrations of GLP-1 than lean or overweight cats [26]. Although the preproglucagon gene encodes for GLP-1 in the gut and glucagon in the pancreas [27], the mechanisms of glucagon secretion and action in FDM are poorly understood. There is some evidence that diabetic cats that underwent remission had a higher glucagon to insulin ratio [28] and that humans with T2DM have hyperglucagonemia [10]. However, whether this dysregulation in glucagon secretion is because of obesity and/or diabetic state is relatively unknown in felines. In addition to the gut, the liver is also being increasingly recognized as an important source of hormones that regulate energy and glucose homeostasis. For example, adropin, a relatively newly discovered peptide hormone, is synthesized and secreted primarily by the liver but is also found in the heart and brain [9]. Dietary macronutrients stimulate adropin secretion and systemic injections of adropin to obese mice have been shown to improve glucose tolerance, skeletal muscle insulin sensitivity and promote weight loss [9, 29]. The role of adropin in the pathophysiology of feline obesity and diabetes is unknown. Therefore, the objective of the present study was to determine whether there were differences in the concentrations of circulating triglycerides, cholesterol, leptin, adiponectin, cytokines, glucagon and adropin in lean, overweight and diabetic cats.

## Methods

Experimental protocols, subject descriptions, feeding protocols, and health assessments have been previously reported for this cohort of cats as part of a study to measure plasma concentrations of GLP-1, glucose-dependent insulinotropic peptide (GIP), insulin and peptide YY (PYY) in client-owned newly diagnosed diabetic cats, and non-diabetic lean or overweight cats [26]. The data on circulating metabolites and hormones that we now provide in the current study have not been previously reported elsewhere.

Briefly, the experimental protocols (AC13–0197) were approved by the University of Calgary Animal Care and Use Committee and the Western College of Veterinary Medicine Animal Research Ethics Board and were conducted in compliance with each university’s ethical guidelines for animal research. Thirty-one cats were recruited from the University of Calgary Veterinary Medicine/Western Veterinary Specialists and Emergency Centre and the Veterinary Medical Center at Western College of Veterinary Medicine through a request for participation. Inclusion criteria were based on body condition score (BCS), the absence of concurrent disease or lack of any medication and acceptance of restraint, venipuncture and standardized diets while exclusion criteria included those that were not amenable to restraint and venipuncture, poor appetites, current history of chronic vomiting or diarrhea, presence of concurrent disease including renal disease or hyperthyroidism, and any current medication administration for any endocrine disorders including, but not limited to, treatment for hyperthyroidism or any medications known to influence glycemia (i.e. glucocorticoids).

### Demographics

The demographics of the study population have been previously reported [26]. Briefly, healthy cats with a BCS of ≤5 were categorized as lean (LC) and those with BCS ≥ 6 as overweight (OC) based on the 9-point BCS [30]. The BCS was assessed independently by experienced investigators (authors CJM, ERS). A total of 10 healthy lean and 11 overweight domestic shorthairs were enrolled in the study. LC included 5 neutered males and 5 spayed females while OC included 6 neutered males and 5 neutered females. Mean age was 8.7 ± 1.1 years in LC and 6.63 ± 0.8 years in OC. Mean body weight was 4.4 ± 0.2 kg in LC and 6.3 ± 0.3 kg in OC while mean BCS was 5 in LC and 7 in OC. Ten diabetic (DC) cats were enrolled in this study wherein 8 were neutered males while 2 were spayed females. Diabetic cats were diagnosed based on client history and clinical signs, hyperglycemia with concurrent glucosuria and elevated fructosamine. DC had a mean age of 10.6 (range 5–15) years, body weight of 5.7 ± 0.5 kg and BCS of 7 (3 lean, 7 overweight).

### Feeding protocol

The feeding protocol was described in full detail previously [26]. Briefly, to eliminate the effects of varying nutrition contents of different feline diets, LC and OC were fed a standardized diet composed of a combination of dry (Adult Optimal Care®, Hill’s Science Diet®, Topeka, KS) and canned (Adult Gourmet Turkey Entrée, Hill’s Science Diet®) 2 weeks prior to the baseline hormone assessments. Following the baseline assessment, LC and OC were exclusively fed a combination of dry and canned high protein diet commonly prescribed to diabetic cats (Prescription Diet® m/d®, Hill’s Science Diet®) until the 4-week assessment. To avoid multiple dietary changes in a feline patient with a newly diagnosed chronic illness, DC were not transitioned to the standardized diet and had baseline assessments performed while on the diets being fed at the time of diagnosis and were then immediately transitioned to the same diabetic diet fed to LC and OC and started on exogenous insulin therapy.

### Blood sampling and health assessment

Blood collection was performed as described previously [26]. Briefly, sampling was performed after a 10-h fast in LC and OC after 2 weeks on the standardized diet (baseline) and then at 4 weeks on the diabetic diet. In DC, blood was also collected after a 10-h fast at baseline and then at 4 weeks on the diabetic diet. For all animals, a complete history was taken at each assessment point including owners’ compliance with diet and fasting recommendations. At the time of each evaluation, animals had a complete physical examination which included body weight and BCS assessments.

### Diabetic management and insulin therapy

Insulin therapy was carried out as previously reported [26]. Briefly, DC were treated with glargine (Lantus®, Sanofi-Aventis, Laval, QC) twice a day at starting dose of 0.25 ± 0.02 U/kg during the morning and 0.23 ± 0.02 U/kg in the evening - with dosages adjusted upon the recommendation of the attending clinician. DC were evaluated routinely for ongoing management of their FDM, however, only preselected time points were used to collect study data. At each recheck, a thorough history was taken. Owners were specifically questioned about clinical signs that could be used as subjective measures of improvement in hyperglycemia, including decreases in polyuria, polydipsia, and polyphagia. DC also had blood glucose curve performed and serum fructosamine concentrations measured at each sampling point. Clinical signs, physical examination parameters, and blood glucose assessments were used to make therapeutic decisions regarding insulin dosage adjustments.

### Measurement of plasma hormone and metabolite concentrations

Fasting plasma concentrations of leptin, adiponectin, glucagon and adropin were measured at baseline and at 4 weeks using commercially available ELISA kits. Only fasting samples were analyzed because it has previously been shown that postprandial concentrations of leptin and adiponectin in cats are not statistically different from fasting samples [31]. Similarly, since glucagon concentrations are highly influenced by circulating insulin concentrations [10], with special consideration to our insulin-treated DC, only fasting blood samples are analyzed. In this case, exogenous insulin would not have been administered to DC for 12 h prior to sampling. In addition, only fasting adropin concentrations were measured as it has been reported in humans that adropin concentrations do not dramatically change after consumption of high sugar or fat meals [32]. Plasma concentrations IL-1b, IL-6, IL-12, MCP-1, and TNFα were determined using Milliplex® ELISA by an independent laboratory (Eve Technologies, Calgary, AB). Each sample was assayed in duplicate following the manufacturer’s recommended protocols. All assays underwent validation procedures using pooled cat plasma. Fasting serum triglyceride, cholesterol, liver enzymes and other analytes (Alkaline phosphatase (ALP), Alanine aminotransferase (ALT), Gamma-glutamyl transferase (GGT), Glutamate dehydrogenase (GLDH) and other analytes (Blood urea nitrogen (BUN), albumin, bilirubin) and fructosamine concentrations were measured using standard laboratory protocols at Prairie Diagnostic Services (Saskatoon, SK).

To minimize the effect of inter-assay variability, the samples were distributed so that each plate received an approximately equal number of samples from each treatment group. Inter-assay CV was assessed by running pooled cat plasma in duplicate on each plate.

Leptin was measured at baseline and at the 4-week assessment point using a feline leptin-specific assay with a range of 0–125 nmol/L (FEE0445, Biotang, Lexington, MA). The intra-assay and interassay CV’s were 12 and 24%, respectively and the assay sensitivity was 30 pmol/L. Spikes of 15.6 and 31.3 nmol/L of leptin in pooled cat plasma yielded recoveries of 101 and 122%, respectively.

Adiponectin was measured at baseline and at the 4 week assessment point with an assay that cross-reacts with human, canine, hamster, monkey, feline and rat adiponectin; with a range of 3–694 nmol/L (RD191023100, BioVendor, Brno, Czech Republic), and was previously validated for feline samples [19]. The intra-assay CV for was 5%, interassay CV was 24%, and assay sensitivity was 3 nmol/L. Spikes of 30 nmol/L to pooled cat plasma yielded a recovery of 100%.

Glucagon concentration was measured using an assay that cross-reacts with human, rat and mouse glucagon with a range of 1.5–120 pmol/L (10–1271-01, Mercodia, Uppsala, Sweden). The intra-assay CV was 5.6%; interassay CV was 3.2%, and assay sensitivity was 1.7 pmol/L. Spikes of 9.06 and 30.5 pmol/L of glucagon to pooled cat plasma resulted in 101 and 105% recoveries, respectively. Linear regression of expected versus measured concentrations for glucagon from serially diluted plasma (1:2 to 1:8) in independent assays yielded average slope of 1.07, *R* ^2^ values (*P* < 0.001) of 0.99, and Y-intercept of −3.36.

Adropin concentration was measured using an assay that cross-reacts with human, mouse and rat adropin, and has a range of 2–20,000 pmol/L (EK-032-35, Phoenix Pharmaceuticals, Burlingame, CA). The intra-assay CV was 14.0%, interassay CV was 0.9% and assay sensitivity was 652 pmol/L. Spikes of 200 and 2000 of adropin to pooled cat plasma yielded recoveries of 86 and 82%, respectively. Linear regression of expected versus measured concentrations for adropin in serially diluted (1:2 to 1:8) pooled cat plasma yielded average slope of 0.66, R^2^ values (*P* < 0.001) of 0.92 and Y-intercept of 23.10.

Plasma concentrations of IL-1b, IL-6, IL-12, MCP-1 and TNFα were measured using a customized feline cytokine assay that utilizes Multiplexing LASER Bead Technology. The intra-assay CV’s, inter-assay CV’s and recoveries for IL-1b were 10, 35 and 32%, respectively, IL-6 were 28, 28 and 34%, respectively, IL-12 were 5, 9 and 112%, respectively, MCP-1 were 31, 35 and 42%, respectively and TNFα were 20, 52 and 32%, respectively. Data for IL-1b, IL-6, MCP-1 and TNFα were not considered for further analyses due to poor assay performance. Only data for IL-12 were analyzed and reported.

### Statistics

Data on LC, OC and DC are reported as mean ± SE and were analyzed using IBM SPSS® v20 (New York, USA). The data for all hormones were log-transformed prior to analyses to improve normality and then analyzed by repeated measures linear mixed models using age and gender as covariates, group (LC, OC, and DC) as between subject factor, week (baseline and 4 week) as within group factor, and group × week interaction. Age and gender were subsequently removed from the overall model as they were not significant. Cats within the group were the random variable on which repeated measures were taken and covariance structures modeled. The covariance structure of the repeated measurements for each variable was modeled either as compound symmetry, heterogeneous compound symmetry, autoregressive, heterogeneous autoregressive, first order antedependence or Toeplitz based on the smallest values of fit statistics for Akaike’s and Bayesian information criteria. The between-group differences for each week were analyzed using ANCOVA with age and gender as covariates followed by Bonferroni test to separate means. The within-group differences between baseline and 4-week hormone concentrations were analyzed with paired *t*-test. Pearson correlation analyses were done to assess the relationship among variables. Significance was set at *P* ≤ 0.05 and trends at *P* < 0.10.

## Results

### Body weight and BCS

As we previously reported [26], at 4 weeks, the OC (6.4 ± 0.3 kg) and DC (5.8 ± 0.5 kg) were significantly heavier than LC (4.4 ± 0.2 kg). Further, the OC (7.0 ± 0.2) and DC (7.0 ± 0.8) had greater BCS than LC (5 ± 0.1) at 4 weeks.

### Glucose, insulin, triglycerides, cholesterol and liver enzymes

**Fig. 1:**
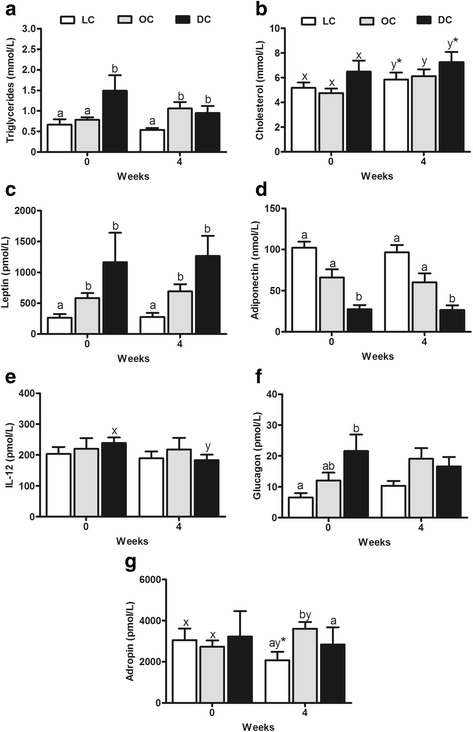
Concentrations of serum triglycerides (**a**), cholesterol (**b**), and plasma leptin (**c**), adiponectin (**d**), IL-12 (**e**), glucagon (**f**) and adropin (**g**) in lean (*n* = 10), overweight (*n* = 11) and diabetic (*n* = 10) cats at baseline and after 4 weeks of feeding a commonly prescribed feline diabetic diet. a,b denotes differences between groups at each week; x,y denotes differences between baseline and 4 weeks (*P* < 0.05). * denotes a trend (*P* < 0.10). Values are mean ± SEM

Fasting blood glucose and plasma insulin were previously reported [26] and are provided here together with serum liver enzymes, BUN, albumin and bilirubin in Table 1. For serum triglyceride concentrations (Fig. 1a), the main effect of group was significant (*P* < 0.05), group x week interactions (*P* = 0.09) tended to be significant, whereas week (*P* = 0.21) was not significant. At baseline, DC had greater triglyceride concentrations by 117 and 96% compared to LC and OC, respectively. At 4 weeks, triglycerides were significantly increased in OC and DC by 63 and 73% compared to LC, respectively.

**Table 1:** Fasting blood glucose, plasma insulin and serum liver enzymes concentrations in lean, overweight and newly-diagnosed diabetic cats at baseline

	Reference	Lean	Overweight	Diabetic
Glucose (mmol/L)	3.5–8.1	3.99 ± 0.16	4.45 ± 0.46	21.07 ± 1.25
Insulin (pmol/L)	N.A.	65.20 ± 16.43	80.92 ± 23.58	47.38 ± 15.20
^a^ALP (U/L)	11–56	27.12 ± 2.15	24.82 ± 2.20	55.70 ± 5.56
^b^ALT (U/L)	30–120	66 ± 6.82	63.09 ± 7.96	90.60 ± 25.43
^c^GGT (U/L)	0–6	0.25 ± 0.16	0 ± 0	0.20 ± 0.13
^d^GLDH (U/L)	1–5	2 ± 0.37	2.64 ± 0.43	23.56 ± 14.65
^e^BUN (mmol/L)	6–11.4	8.77 ± 0.89	9.74 ± 0.53	8.30 ± 0.56
Albumin (g/L)	27–39	37.88 ± 1.57	38.09 ± 0.80	37.90 ± 1.15
Bilirubin (μmol/L)	0–2	1.35 ± 0.35	1.49 ± 0.22	1.53 ± 0.15

Values are expressed as mean ± SEM, *n* = 10–11/group

^a^Alkaline phosphatase

^b^Alanine aminotransferase

^c^Gamma-glutamyl transferase

^d^Glutamate dehydrogenase

^e^ Blood Urea nitrogen

For cholesterol concentrations (Fig. 1b), the main effect of group (*P =* 0.17) and group x week interactions (*P* = 0.22) were not significant, whereas week (*P* < 0.01) was significant. From baseline to 4 weeks within groups, the transition to the standardized diet commonly prescribed to diabetic patients resulted in significantly increased circulating cholesterol concentrations in LC by 11%, OC by 27% and in DC by 13%.

### Leptin, adiponectin, and IL-12

For plasma leptin concentrations (Fig. 1c), the main effect of group was significant (*P* < 0.01), whereas week (*P* = 0.70) and group x week interactions (*P* = 0.98) were not. At baseline, leptin concentrations in OC and DC were increased by 172 and 221% compared to LC, respectively. At 4 weeks, plasma leptin concentrations in OC and DC were increased by 188 and 214% compared to LC, respectively. Overall, leptin concentrations were positively correlated with BCS (*r* = 0.43, *P* < 0.01) and triglycerides (*r* = 0.29, *P* = 0.02).

The main effect of group was significant (*P* = 0.01) for plasma adiponectin (Fig. 1d), with a trend for week (*P* = 0.07) but no significant group x week interaction (*P* = 0.81). At baseline, DC had 61 and 45% lower adiponectin than LC and OC, respectively. At 4 weeks, circulating concentrations of adiponectin were significantly lower in DC by 81 and 69% compared to LC and OC, respectively. Overall, adiponectin concentrations were negatively correlated with BCS (*r* = −0.29, *P* = 0.03) and triglycerides (*r* = −0.32, *P* = 0.02).

No significant difference was observed in the plasma concentrations of IL-12 (Fig. 1e) across groups (*P* = 0.87). There was a trend for group x week interaction (*P* = 0.07) with plasma IL-12 concentrations decreasing by 25% from baseline to 4 weeks in DC.

### Glucagon and adropin

There was a significant group effect for circulating glucagon (Fig. 1f) concentrations (*P* = 0.01) but none for week (*P* = 0.14) and group x week interactions (*P* = 0.12). At baseline, DC had increased glucagon compared to LC by 236% but not compared to OC. No differences were observed at 4 weeks. Glucagon concentrations were positively correlated with triglycerides (*r* = 0.30, *P* = 0.03) and cholesterol (*r* = 0.26, *P* = 0.05).

For plasma adropin concentrations (Fig. 1g), there were significant effects of group (*P* = 0.03) and group x week interactions (*P* < 0.01). At baseline, no difference was observed among groups. However, at 4 weeks, OC had increased adropin concentrations compared to LC and DC by 112 and 79%, respectively. From baseline to 4 weeks within each group, adropin was significantly increased by 35% in OC but tended to be decreased by 34% in LC. Overall, adropin concentrations were positively correlated with BCS (*r* = 0.27 *P* = 0.05).

## Discussion

We compared the circulating concentrations of triglycerides, cholesterol, leptin, adiponectin, IL-12, glucagon and adropin in client-owned lean, overweight and diabetic cats. Our results highlight several important findings. First, serum concentrations of triglycerides were greater in diabetic at baseline and were increased in both diabetic and overweight cats at 4 weeks. Second, plasma leptin concentrations were greater in diabetic and overweight cats at baseline and 4 weeks, whereas adiponectin was lower in diabetic compared to lean and overweight cats at baseline and 4 weeks after starting the cats on the high protein/low carbohydrate standardized diet. Third, diabetic cats had greater baseline plasma glucagon concentrations compared to lean cats, lower adropin than overweight cats at 4 weeks, and lower IL-12 concentrations at 4 weeks than baseline.

The hypertriglyceridemia in the OC and DC is consistent with other reports demonstrating that increased circulating triglycerides are seen in obese [24] but not lean cats [33]. Similar to humans, plasma lipids are also elevated in T2DM [34]. Feeding the commercially available high protein and low carbohydrate diet commonly prescribed to diabetics did not affect triglycerides but increased cholesterol in all groups. To our knowledge, the effect of high protein/low carbohydrate diets on cholesterol in OC and DC are not yet reported. In kittens, high protein/low carbohydrate diets did not change cholesterol concentrations [35]. Cholesterol also did not differ between healthy adult cats fed with a moderate protein/high carbohydrate diet and high protein/low carbohydrate diet at comparable fat contents [36]. Because the dietary macronutrient composition of the m/d diet in our study is in general comparable to other reports, it is less likely that the increased cholesterol in OC and DC are primarily of dietary origin. However, whether the dietary factors interact with the host to predispose OC and DC to enhanced cholesterogenesis remain to be determined.

The circulating leptin concentrations reflected adiposity and were significantly elevated in DC and OC compared with LC at baseline and 4 weeks. Our results are in agreement with other studies supporting the correlation with adiposity with increased leptin concentrations in overweight and obese cats and increases in cats undergoing weight gain [19, 37]. Similar to other studies [31, 38], fasting leptin concentrations were also not affected by feeding a high protein diet which is likely due to the maintenance of a stable body weight in our subjects.

We found that DC had significantly decreased adiponectin compared to OC and LC throughout the study. The effects of adiposity on circulating total adiponectin concentrations are controversial with some studies indicating that OC have decreased adiponectin concentrations [14–18], whereas others failed to detect differences between LC and OC in total adiponectin [19, 20]. In our study, total adiponectin concentrations across all groups showed weak negative correlation with BCS. Similarly, in humans with T2DM, adiponectin concentrations are greatly reduced compared to obese humans [39]. Importantly, we also noted a reduction of adiponectin concentrations in DC compared to both LC and OC, suggesting that decreased adiponectin is also present in FDM. The lower adiponectin concentrations in DC might have contributed to the lower negative correlation of BCS with adiponectin despite comparable BCS between the OC and DC.

Previous rodent [40, 41] and human [42, 43] studies reported that pro-inflammatory cytokine IL-12 is increased during obesity and T2DM; however, there is limited data in cats. Hoenig et al. [21, 44] reported an increase in the mRNA abundance of TNF-α in white adipose but without differences in plasma concentrations of TNF-α, IL-1, and IL-6 of OC compared to LC. Van de Velde et al. [22] reported increased mRNA abundance of TNF-α, IFN-ϒ, chemokine ligand 5 (CCL-5) and MCP-1 in adipose tissue of OC compared to LC but did not detect differences in IL-6 and IL-10. It is unclear whether the alterations in mRNA abundance of cytokines in adipose tissues are reflective of circulating cytokine concentrations in cats. In our study, despite the feline-specific nature of the multiplex assay for cytokines, the poor assay performance (< 50% recoveries) of IL-1b, IL-6, MCP-1, and TNFα precluded their assessment in our patient population. However, the recoveries and assay CV’s for IL-12 were acceptable. We noted that IL-12 did not differ between OC and LC but decreased in DC. Given that IL-12 secretion is increased in T2DM [40–43], whether the reduction in IL-12 in DC at 4 wks is due to improvements brought about by exogenous insulin administration or dietary change from baseline remains to be addressed.

At baseline, prior to exogenous insulin therapy, DC had greater plasma glucagon concentrations compared to LC. Previous research did not find a statistical difference in the number of immunoreactive α-cells between DC and non-DC [23] but Tschour et al. [28] reported a higher ratio of glucagon to insulin in FDM that remitted versus those that did not. In humans, increased circulating concentration of glucagon is an indicator of insulin resistance and diabetic status [45]. Our results also support the hypothesis that glucagon is involved in FMD pathophysiology; whether the lack of differences in plasma glucagon concentrations at 4 weeks is a consequence of an insulin-induced downregulation of glucagon secretion from exogenous insulin therapy in DC remains to be demonstrated.

For the first time, we report on plasma concentrations of adropin in cats. At baseline, adropin concentrations did not differ between our study groups. However, we found that adropin concentrations were increased in OC but not LC or DC following 4 weeks. The association of adropin with diabetes is controversial. In rodents, increased adropin immunoreactivity was noted particularly in the pancreas, liver and kidneys of streptozotocin diabetic rats [46], whereas adropin concentrations were found to be lower in women with gestational diabetes [47–49] and exogenous adropin improves glucose tolerance and insulin action in mice [29]. Interestingly, in our study, despite comparable BCS and diets, adropin concentrations were lower in DC than OC; whether this is due to diabetic status and/or exogenous insulin therapy remains to be determined.

## Conclusions

Our results suggest that overweight and diabetic cats are characterized by hypertriglyceridemia and hyperleptinemia; however, reduced adiponectin and adropin in diabetics are disassociated from overweight cats. Thus, feline obesity and diabetes are associated with differential circulating concentrations of adiponectin and adropin. Whether the differential circulating concentrations of these hormones are due to dysregulations in the tissue expression, secretion, action and/or elimination remain to be determined in cats with varying degrees of body condition and diabetic status.

## Abbreviations

ALP: Alkaline phosphatase
ALT: Alanine aminotransferase
ANCOVA: Analysis of covariance
BCS: Body condition score
BUN: Blood urea nitrogen
CCL-5: Chemokine ligand 5
CV: Coefficient of variation
DC: Diabetic cats
ELISA: Enzyme-linked immunosorbent assay
FDM: Feline diabetes mellitus
GGT: Gamma-glutamyl transferase
GIP: Glucose-dependent insulinotropic peptide
GLDH: Glutamate dehydrogenase
GLP-1: Glucagon-like peptide-1
IFNϒ: Interferon gamma
IL-12: Interleukin-12
IL-1B: Interleukin-1B
IL-6: Interleukin-6
LC: Lean cats
MCP-1: Monocyte chemoattractant protein-1
OC: Overweight cats
PYY: Peptide YY
T2DM: Type 2 diabetes mellitus
TNFα: Tumor necrosis factor alpha
